# Heterogeneous Nuclear Ribonucleoprotein R Cooperates with Mediator to Facilitate Transcription Reinitiation on the c-Fos Gene

**DOI:** 10.1371/journal.pone.0072496

**Published:** 2013-08-13

**Authors:** Aya Fukuda, Miho Shimada, Tomoyoshi Nakadai, Ken Nishimura, Koji Hisatake

**Affiliations:** 1 Faculty of Medicine, University of Tsukuba, Tsukuba, Ibaraki, Japan; 2 Laboratory of Biochemistry and Molecular Biology, The Rockefeller University, New York, New York, United States of America; Università degli Studi di Milano, Italy

## Abstract

The c-*fos* gene responds to extracellular stimuli and undergoes robust but transient transcriptional activation. Here we show that heterogeneous nuclear ribonucleoprotein R (hnRNP R) facilitates transcription reinitiation of the c-*fos* promoter *in vitro* in cooperation with Mediator. Consistently, hnRNP R interacts with the Scaffold components (Mediator, TBP, and TFIIH) as well as TFIIB, which recruits RNA polymerase II (Pol II) and TFIIF to Scaffold. The cooperative action of hnRNP R and Mediator is diminished by the cyclin-dependent kinase 8 (CDK8) module, which is comprised of CDK8, Cyclin C, MED12 and MED13 of the Mediator subunits. Interestingly, we find that the length of the G-free cassettes, and thereby their transcripts, influences the hnRNP R-mediated facilitation of reinitiation. Indeed, indicative of a possible role of the transcript in facilitating transcription reinitiation, the RNA transcript produced from the G-free cassette interacts with hnRNP R through its RNA recognition motifs (RRMs) and arginine-glycine-glycine (RGG) domain. Mutational analyses of hnRNP R indicate that facilitation of initiation and reinitiation requires distinct domains of hnRNP R. Knockdown of hnRNP R in mouse cells compromised rapid induction of the *c-fos* gene but did not affect transcription of constitutive genes. Together, these results suggest an important role for hnRNP R in regulating robust response of the *c-fos* gene.

## Introduction

The c-*fos* gene is regulated by a myriad of stimuli to express a large amount of its mRNA in a short period of time [1], [2], [3]. Such regulated transcription requires concerted actions among general transcription factors (GTFs), activators, as well as coactivators, which lead ultimately to the formation of the preinitiation complex (PIC) that consists of RNA polymerase II and six GTFs including TFIIA, TFIIB, TFIID, TFIIE, TFIIF and TFIIH [4], [5]. Whereas activators and GTFs are essential for activated transcription by Pol II, it has become increasingly clear that coactivators play a key role as a regulatory interface that relays signals from activators to the PIC [6], [7], [8]. Additionally, coactivators modify chromatin to render promoters more accessible to the transcriptional machinery, integrate signals from diverse activators and kinases, and coordinate different steps of gene expression from initiation to post-transcriptional processes [6], [7], [9].

Our previous study showed that hnRNP R functions as a coactivator to enhance transcription from the c-*fos* promoter *in vitro*, in conjunction with positive cofactor 4 (PC4) and Mediator [10]. hnRNP R possesses archetypical RNA-binding domains, RRMs and the RGG domain, which are found in many proteins involved in RNA splicing [11]. Although hnRNP R cooperates with other classic coactivators such as PC4 and Mediator to enhance c-*fos* transcription, the presence of RNA-binding motifs suggests a possible role for hnRNP R in post-transcriptional processes that involve the transcript RNA. Indeed, certain coactivators that possess RNA-binding motifs have been shown to play roles in post-transcriptional RNA processing [12], [13]. For instance, peroxisome proliferator-activated receptor-γ coactivator-1 (PGC-1) harbors RNA-binding domains in its C-terminus and has been demonstrated to increases the expression of its target genes by coupling transcription to RNA processing [12]. A coactivator for nuclear receptors (CoAA), an hnRNP-like protein that contains two RRMs, regulates alternative splicing of the transcript in a steroid hormone-specific manner [14]. However, given that hnRNP R enhances transcription in purified *in vitro* transcription system [10], where no apparent posttranscriptional processes occur, it remained to be determined how RNA-binding motif-containing hnRNP R acts as a coactivator to support a high level of c-*fos* transcription *in vitro*.

The hallmark of immediate-early genes such as c-*fos* is a transient but robust transcriptional response to various stimuli. Presumably, to elicit this type of high-level transcription, cells need a mechanism that enables a rapid succession of reinitiations, which allow a single gene to be transcribed by multiple RNA polymerases at the same time. In highly expressed rRNA genes (rDNAs), which are transcribed exclusively by Pol I (Class I genes), a single rDNA unit is densely loaded with, and transcribed by, multiple Pol I molecules as visualized by electron microscopy [15], [16]. This high-density loading of Pol I is achieved, in part, by termination-coupled initiation, in which Pol I molecules that have terminated transcribing one rDNA unit is then transferred directly to the promoter of the downstream rDNA unit [17]. In another type of highly expressed genes such as 5S rRNA and tRNA genes, which are transcribed by Pol III (Class III genes), transcription initiation and termination are tightly coupled within a single gene, so that a Pol III molecule initiates transcription immediately after terminating transcription of the same gene, a process known as hyperprocessive reinitiation [17]. For genes transcribed by Pol II (Class II genes), Mediator has been implicated in facilitating transcription reinitiation by forming a structure termed Scaffold on the promoter [18]. Compared to Class I and Class III genes, however, far less is known about a mechanism that facilitates transcription reinitiation of Class II genes.

We show here that hnRNP R cooperates with Mediator to enhance transcription by facilitating not only initiation but subsequent reinitiations as well. This effect is diminished by the CDK8 module, which consists of CDK8, Cyclin C, MED12 and MED13, within Mediator. hnRNP R shows interactions with TFIIB as well the Scaffold components including Mediator, TBP and TFIIH, raising the possibility that hnRNP R serves as a bridge between the incoming Pol II/TFIIF/TFIIB and the promoter-bound Scaffold. Cell-based knockdown experiments indicate that hnRNP R is required for rapid induction of the *c-fos* gene but is dispensable for constitutively transcribed Class II genes as well as for Class I and Class III genes. These results indicate that hnRNP R plays an important role as a transcriptional regulator during serum induction of the *c-fos* gene.

## Materials and Methods

### Antibodies

An anti-hnRNP R antibody was prepared by immunizing rabbits with purified recombinant His-tagged hnRNP R. Recombinant hnRNP R was induced at 30°C for 3 hrs by 1 mM IPTG as a His-tagged protein in BL21(DE3)pLysS harboring pET15b-hnRNP R and purified from the soluble fraction using TALON® Superflow™ Metal Affiniy Resin (Takara Bio). Partially purified hnRNP R was separated by SDS-PAGE and, after excision from the gel, hnRNP R was eluted from the gel using a Model 422 Electro-Eluter (Bio-Rad Laboratories). The purified recombinant human hnRNP R was used to immunize rabbits, and the obtained anti-hnRNP R sera were affinity purified by an antigen column, in which Glutathione S-transferase (GST)-hnRNP R was cross-linked to Glutathione Sepharose 4B (GE healthcare).

All the other antibodies were purchased commercially. Anti-MED13 rabbit polyclonal antibody (ab76923) and anti-Cyclin C rabbit polyclonal antibody (ab85927) were purchased from Abcam. Anti-MED12 rabbit polyclonal antibody (A300-774A) and anti-CDK8 goat polyclonal antibody (sc-1521) were purchased from Bethyl Laboratories and Santa Cruz Biotechnology, respectively. Anti-Ets-related transcription factor (Elk-1) antibody (Invitrogen, 458700) and anti-CRE-binding factor (CREB) antibody (Cell Signaling Technology, 9104) were purchased from Invitrogen and Cell Signaling Technology, respectively.

### Protein purification and *in vitro* transcription assays

Pol II, TFIIA, TFIIB, FLAG-tagged TFIID (F:TFIID), TFIIE, TFIIF and F:TFIIH were prepared as described previously [19]. Flag-tagged serum response factor (F:SRF), F:Elk-1, F:CREB, Flag-tagged activating transcription factor 1 (F:ATF1), F:hnRNP R and Mediator were purified as described previously [10]. Deletion mutants of hnRNP R were prepared essentially in the same way as wild-type hnRNP R; partial purification by HiTrap SP HP and HiTrap Q HP, followed by affinity purification by ANTI-FLAG® M2-Agarose (Sigma-Aldrich). *In vitro* transcription reactions were performed as described previously [10], [19]. A phosphor image analyzer (BAS-2500, Fujifilm) was used to measure the levels of the transcripts. A PCR-based method was used to create a series of transcription templates possessing the G-free cassette of different lengths.

### RNase protection assays

The RNA probes were designed to contain the 5′ or 3′ region of the G-free cassette (105 nt or 114 nt, respectively) and were labeled by T7 RNA polymerase (New England Biolabs) in the presence of [α-^32^P] UTP. *In vitro* transcription products from the *c-fos* promoter were hybridized with either the ^32^P-labeled 5′ or 3′probe at 70°C for 11 hrs. After digestion by RNase A and RNase T1 at 30°C for 1 hr followed by proteinase K treatment at 37°C for 30 min, the RNA was separated on an 8% denaturing polyacrylamide gel and detected by autoradiography. The T7 promoter-driven transcripts were used as a control.

### Protein-protein and protein-RNA interactions

GST pull-down assays were performed essentially as described [20], except that GST-fusion proteins were expressed and purified from High Five insect cells. For RNA-binding assays, the ^32^P-labeled RNA probe was prepared from the G-free cassette by T7 RNA polymerase in the presence of [α-^32^P] CTP. The labeled RNA probe was incubated at 25°C for 30 min with either GST or GST-hnRNP R immobilized to Glutathione Sepharose 4B in the presence of RNase inhibitor (35 units) and 8 mM MgCl_2_, and the unbound RNA was washed out six times by a fifty times the volume of buffer C (20 mM Hepes-KOH, pH 7.9, 10% glycerol, 1 mM EDTA) containing 100 mM KCl, 1 mM dithiothreitol, 0.5 mM PMSF (phenylmethylsulfonyl fluoride), 0.01% Nonidet P-40. After proteinase K treatment, phenol/chloroform extraction and ethanol precipitation, the isolated RNA was separated on a 5% denaturing polyacrylamide gel and analyzed by autoradiography. For co-immunoprecipitation, HeLa nuclear extract (∼20 mg protein) was incubated with 1 µg of the purified anti-hnRNP R antibody in the presence of protease inhibitor cocktail (Sigma-Aldrich) and 0.1 µM MG132 at 4°C for 2 hrs, and then 20 µl of rProtein A Sepharose Fast Flow (GE Healthcare) was added. After incubation at 4°C for 1 hr, the sepharose was washed with the buffer C containing 100 mM KCl, 0.5 mM PMSF and 0.01% Nonidet P-40 to remove unbound proteins. The bound proteins were analyzed by western blotting using the antibodies indicated in the figure. For RNA binding, the immunoprecipitated RNA was isolated by Sepasol®-RNA I Super G (Nacalai tesque) and analyzed by RT-PCR.

### Cell culture and RNAi experiments

Mouse fibroblast C3H 10T1/2 cells were obtained from the RIKEN BRC through the National Bio-Resource Project of the MEXT, Japan. The cells were cultured at 37°C under 5% CO_2_ in Eagle’s basal medium (BME) supplemented with 10% fetal bovine serum (FBS) and 0.03% L-glutamine. For hnRNP R knockdown, 1×10^5^ cells were cultured on a 6-cm plate at 37°C overnight and were transfected with 30 pmole of Stealth RNAi™ siRNA for hnRNP R (Invitrogen, MSS232544) in the presence of lipofectamine™ RNAiMAX. Stealth RNAi Negative Control with a similar GC content (Low GC Duplex #2, Invitrogen) was used as a negative control. After 24-hr culture of the cells at 37°C, the medium was replaced with fresh BME containing 0.5% FBS and 0.03% L-glutamine, and the cells were allowed to grow at 37°C for 24 h. Serum stimulation of the cells were performed by replacing the medium with fresh BME containing 20% FBS and 0.03% L-glutamine, and total cellular RNAs were isolated from the collected cells by using Sepasol®-RNA I Super G (Nacalai tesque). First-strand cDNAs were prepared by PrimeScript® II 1st strand cDNA Synthesis Kit (Takara Bio) according to the manufacturer’s instruction. Quantitative PCR (qPCR) was performed using the 7500 Fast Real-Time PCR System (Applied Biosystems). The primer sets used for qPCR are shown in supplemental table S1.

## Results

### hnRNP R facilitates transcription reinitiation

**Figure 1:**
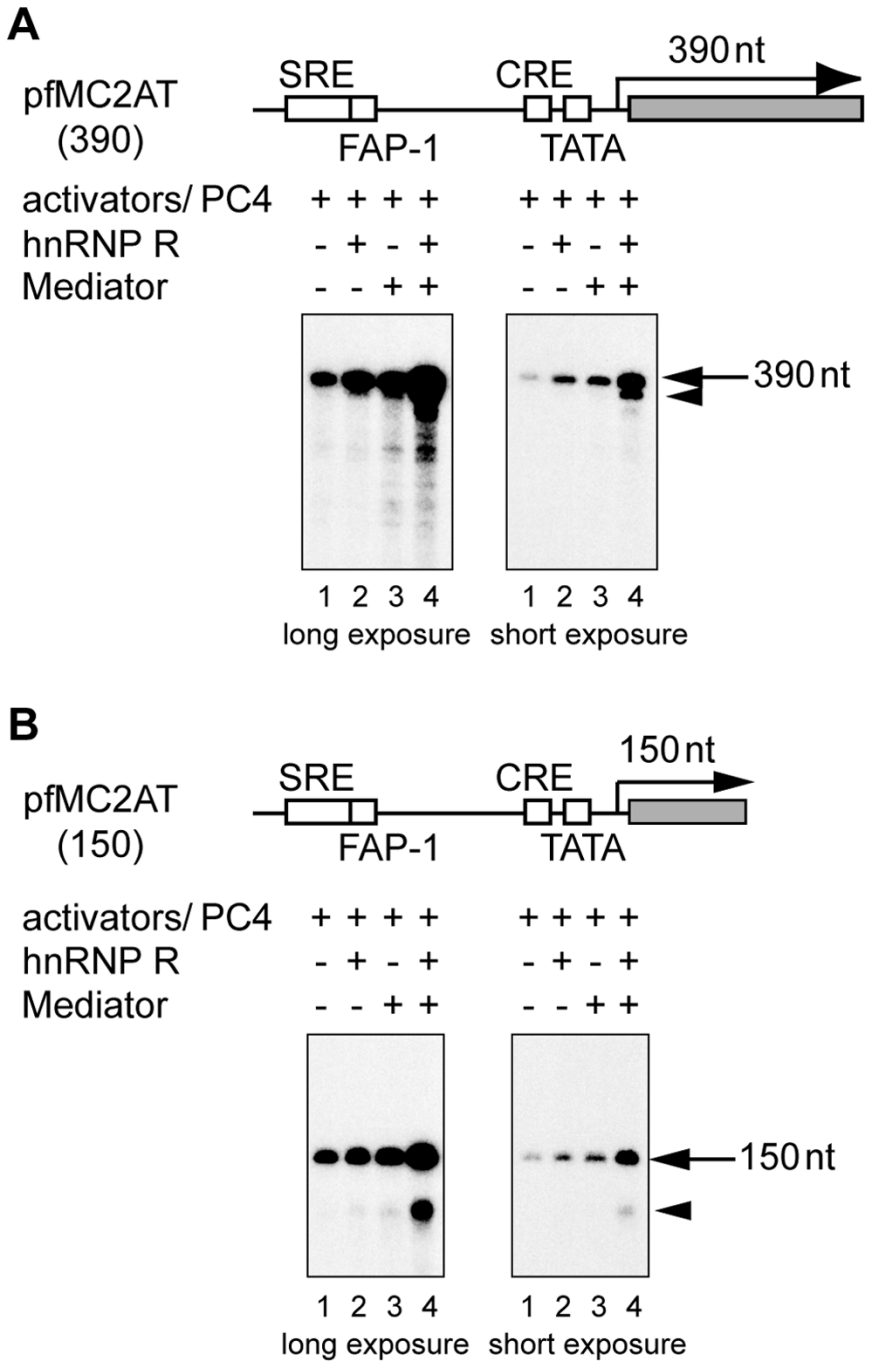
hnRNP R and Mediator enhance transcription from the c-*fos* promoter in a cooperative manner. (A) *In vitro* transcription was performed in a reaction containing TFIIA (10 ng), TFIIB (10 ng), FLAG-tagged TFIID (F:TFIID) (which contained ∼0.1 ng of TBP), TFIIE (10 ng), TFIIF (20 ng), TFIIH (∼20 ng), Pol II (∼100 ng), F:SRF (20 ng), F:Elk-1 (10 ng), F:CREB (20 ng), F:ATF1 (20 ng) and PC4 (100 ng) in the presence (+) or absence (–) of hnRNP R (100 ng) and 3xF:Mediator (∼20 ng) as shown in the figure. pfMC2AT (100 ng), which harbors the c-*fos* promoter and the 390-nt G-free cassette, was used as a template. The reaction was performed in the presence of 0.2 mM ATP, 0.2 mM UTP, 12.5 µM CTP, 0.1 mM 3′-O-methyl GTP and 10 µCi of [α-^32^P] CTP. The arrow indicates the position of the 390-nt transcript (lanes 1–4), and the arrowhead indicates the shorter transcript observed in the presence of both Mediator and hnRNP R (lane 4). SRE, FAP-1, CRE and TATA indicate the serum response element, *c-fos* AP-1 site, cAMP-responsive element and TATA box, respectively. (B) *In vitro* transcription was performed as described in (A), except that the reaction contained the template, pfMC2AT(150), which harbors the 150-nt G-free cassette instead of the 390-nt G-free cassette. The arrow and arrowhead indicate the positions of the 150-nt transcript and the shorter transcript, respectively.

Using reconstituted *in vitro* transcription system, we observed that hnRNP R cooperates with Mediator and PC4 to enhance transcription from the c-*fos* promoter by ∼100 fold over the basal level [10]. Whereas activators and PC4 activated transcription by ∼10-fold, addition of hnRNP R and Mediator further enhanced transcription by ∼12-fold [10] (Figure 1A, lanes 1 versus 4). In the transcription reaction in the presence of hnRNP R and Mediator, the transcript produced from the 390-nt G-free template (pfMC2AT) always appeared to extend downward (Figure 1A, long exposure, lane 4). Indeed, shorter exposure of the same gel revealed another transcript slightly shorter than the 390-nt transcript (Figure 1A, short exposure, lane 4). Using a 150-nt G-free cassette to perform the same transcription assays, we observed a similar pattern of two transcripts that were separated more clearly (Figure 1B, lanes 1–4). In both assays using 390-nt and 150-nt G-free cassettes, the differences in length between the full-length and shorter transcripts were ∼30 nt (Figure 1A and 1B, lane 4), which corresponds to the length of DNA covered by an elongating Pol II [21]. Therefore, we reasoned that the shorter transcript might be produced from reinitiation, as demonstrated elegantly by the colliding polymerases reinitiation assays [22]. In this report, it was shown that Pol IIs arrested by the preceding Pol II at the end of the G-free cassette produce a transcript ∼30-nt shorter than that by the preceding Pol II [22].

**Figure 2:**
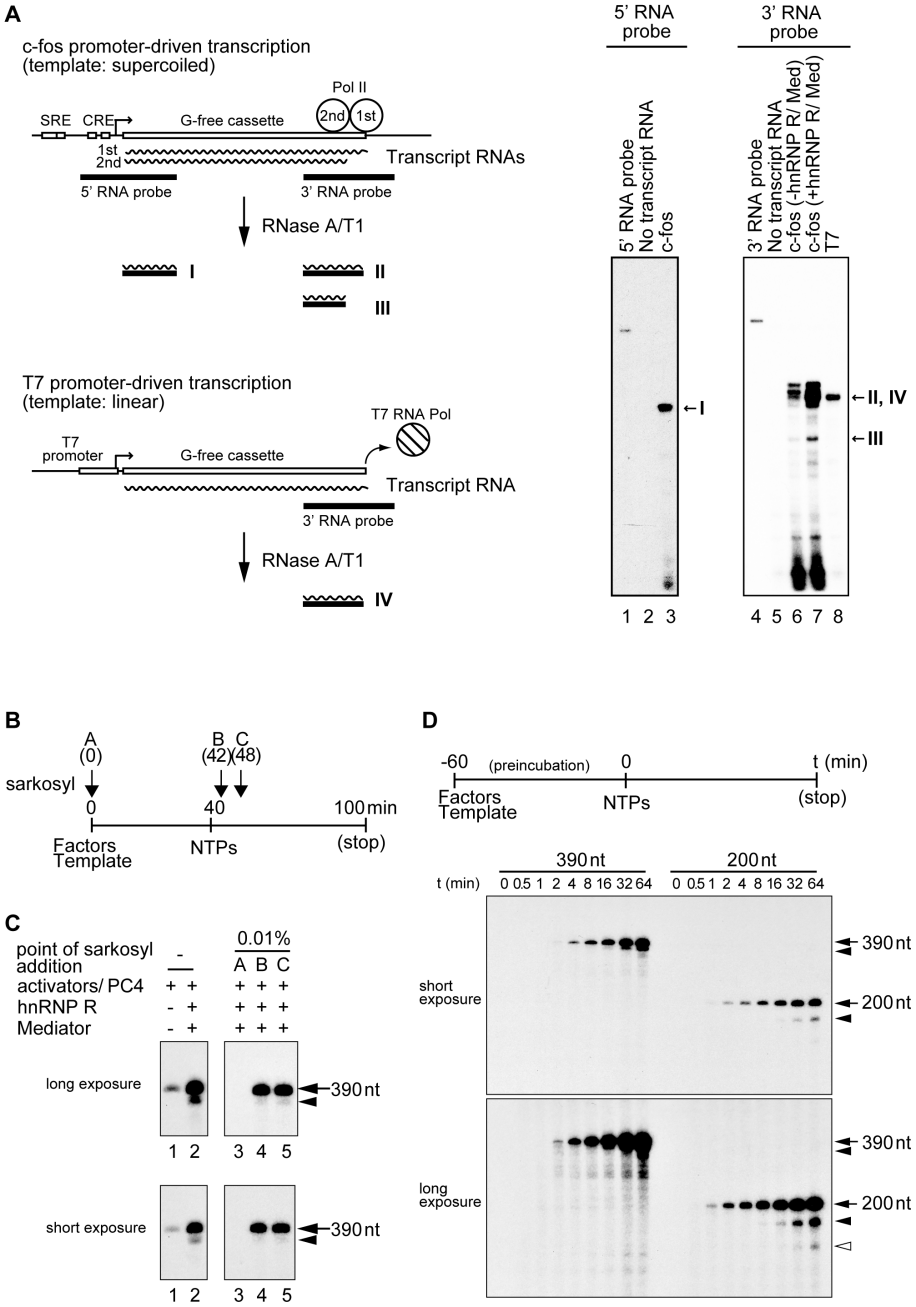
hnRNP R facilitates transcription reinitiation in the presence of Mediator. (A) RNase protection assays were performed using the ^32^P-labeled RNA probes against the 5′ or 3′ region of the G-free cassette. After hybridization of each ^32^P-labeled probe and the transcripts, the products were treated by RNase A and T1 to remove the unhybridized regions. The digested products were separated on an 8% denaturing polyacrylamide gel and detected by autoradiography. As depicted schematically, the first- and second-round transcripts are transcribed by the Pol II system from the supercoiled *c-fos* template. The two *c-fos* transcripts produce a single band (I) when the 5′ probe is used for the RNase protection assay while they produce two bands (II and III) when the 3′ probe is used. By contrast, a single control transcript is transcribed by T7 RNA polymerase from the linear control template. This T7 promoter-based transcript produces a single band (IV) even when the 3′ probe is used for the same RNase protection assay. The results of the RNase protection assays are shown in the right panels, and the positions of the expected products are indicated on the right (I, II, III and IV). The used probe (5′ or 3′ RNA probe) is indicated on the top and loaded in lane 1 or 4. No transcript RNA indicates that the 5′ or 3′ RNA probe was treated by RNaseA/T1 without hybridization with any transcript RNAs, and c-fos (-hnRNP R/Med) or c-fos (+hnRNP R/Med) indicates the transcripts from *c-fos* promoter-driven transcription in the absence or presence of hnRNP R and Med(0.85), respectively. T7 indicates the transcript produced from T7 promoter-driven transcription. (B) GTFs, Pol II, the four activators (SRF, Elk-1, CREB and ATF1), PC4 and the template pfMC2AT(390) were incubated at 30°C for 40 min to form preinitiation complex, and then ATP, CTP, UTP and 3′-*O*-methyl-GTP (NTPs) were added to initiate transcription. After additional 60-min incubation, 20 mM EDTA pH 8.0 and 0.2% SDS were added to stop the reaction. hnRNP R and 3xF:Mediator were added as indicated in the figure. 0.01% Sarkosyl was added before preinitiation complex formation (A: 0 min) or immediately after initiation of transcription (B: 42 min, C: 48 min). (C) The transcripts from the reactions were separated on a 5% denaturing polyacrylamide gel and detected by autoradiography. The positions of the 390-nt transcript (arrow) and the second-round transcript (arrowhead) are indicated on the right. (D) Time-course analyses of the reinitiation products were performed using the pfMC2AT (390) or pfMC2AT (200) template. t indicates the incubation time (min) after the addition of the nucleotides. The upper panel shows the outline of the experiment. The positions of the first- (arrow), second- (arrowhead) and third-round transcripts (white arrowhead) are indicated on the right.

We verified the nature of the shorter transcripts by RNase protection assays as shown in Figure 2A. When the RNA probe against the 5′ region of the G-free cassette was used, only a single band was detected, indicating that the initiation from a cryptic promoter does not occur in our *in vitro* transcription system. On the other hand, the use of the RNA probe against the 3′ region of the G-free cassette detected two bands in the presence of hnRNP R and Mediator (Figure 2A, lane 7), suggesting that the shorter transcript lacks part of the 3′ end of the G-free cassette as we expected. To confirm that the shorter transcript is indeed the second-round transcript, we used an assay in which transcription reinitiation could be blocked by Sarkosyl. Sarkosyl is an anionic detergent that inhibits distinct steps of transcription depending on its concentration [23]. When 0.01% Sarkosyl is added to the transcription reaction *in vitro*, it inhibits initiation but allows elongation by Pol II [24]. Therefore, if added immediately after initiation of transcription, 0.01% Sarkosyl specifically inhibits subsequent reinitiation. As shown in Figure 2B, the factors and template were pre-incubated for 40 min prior to the addition of four nucleotides (ATP, UTP, CTP, 3′-*O*-methyl-GTP), and 0.01% Sarkosyl was added at the indicated time points. When 0.01% Sarkosyl was added at 0 min, no transcript was observed (Figure 2C, lane 3) because PIC formation is inhibited by this concentration of Sarkosyl [24]. Addition of 0.01% Sarkosyl immediately after initiation of transcription (42 min or 48 min) did not affect the full-length transcript but virtually eliminated the shorter transcript, indicating that the shorter transcript is indeed derived from reinitiation (Figure 2C, lanes 4 and 5). It is of note that the reinitiation is enhanced to very small extents by either hnRNP R or Mediator alone, but to a much larger extent when both hnRNP R and Mediator are added to the reactions (Figure 1A and 1B), indicating that both hnRNP R and Mediator are required to enhance reinitiation.

To further corroborate the above findings, we examined the appearance of the shorter transcripts during the course of the transcription reaction. After a 60-minute pre-incubation to form the PIC, four nucleotides were added to the transcription reaction, which was then allowed to proceed for indicated periods of time (Figure 2D, t). As shown in Figure 2D, the shorter transcript appeared ∼15 min later than the full-length transcript. Indeed, if the incubation is allowed for 64 min (∼124 min after the start of incubation), a transcript that corresponds to the third round of transcription started to appear (Figure 2D, long exposure). Taken together, these results demonstrate that hnRNP R and Mediator cooperate to facilitate transcription reinitiation from the c-*fos* promoter *in vitro*.

### Mediator containing CDK8 and Cyclin C does not facilitate transcription reinitiation

Previous studies demonstrated that Mediator is crucial for transcription reinitiation by forming a stable structure on the promoter, termed Scaffold that consists of TFIIA, TFIID, TFIIE, TFIIH and Mediator [18]. One of the Mediator subunits, CDK8, which constitutes the CDK8 module together with Cyclin C, MED12 and MED13, has been shown to repress transcription [25], [26], [27] as well as transcription reinitiation [28]. However, other studies provide evidence that CDK8 plays a positive role in regulating transcription [29], [30], [31], [32]. In light of the dual roles of CDK8 and, by extension, the CDK8 module, in regulating transcription, we wished to know if the CDK8 module regulates hnRNP R-mediated transcription reinitiation.

**Figure 3:**
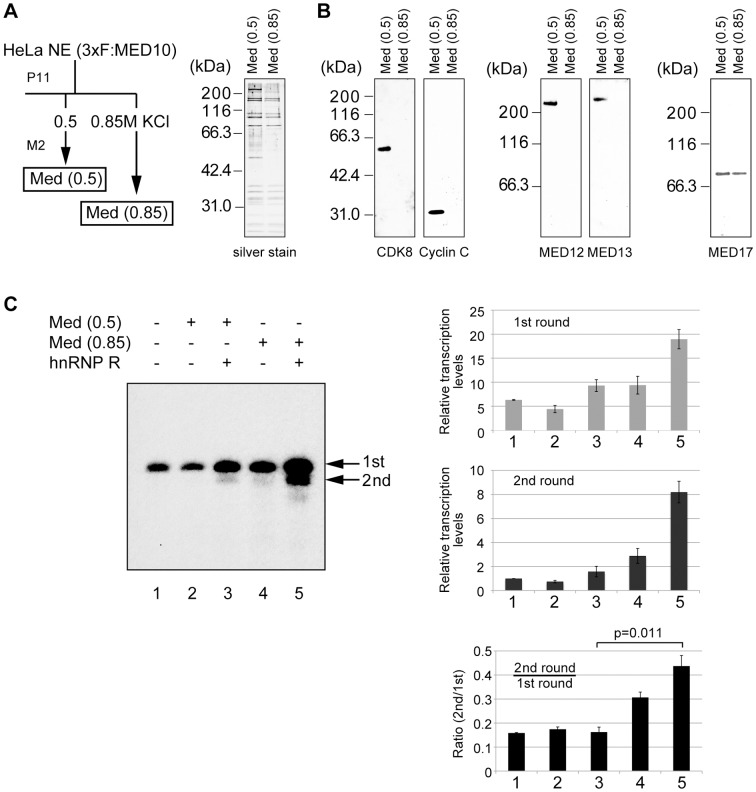
The Mediator containing the CDK8 module does not facilitate transcription reinitiation. (A) Med(0.5) and Med(0.85) were purified from HeLa nuclear extract expressing 3xFLAG-tagged MED10 by using phosphocellulose (P11) and anti-FLAG® M2 affinity gel. The purified Med(0.5) and Med(0.85) were separated by SDS-PAGE and silver-stained. The positions of the molecular mass marker are indicated on the left. Mediator was detected via 3xFLAG-tagged MED10 using an anti-FLAG antibody. (B) Western blot analyses were performed using anti-CDK8, anti-Cyclin C, anti-MED12, anti-MED13 or anti-MED17 antibody. (C) *In vitro* transcription was performed as described in Figure 1A. hnRNP R, Med(0.5) and Med(0.85) were added as shown in the figure. The arrows indicate the first-round (1st) and second-round (2nd) transcripts, and the values (means ± SE) of the quantified data from four independent experiments are shown in the top (1st round) and middle (2nd round) panels on the right. The bottom panel on the right shows the ratios of the second-round to first-round transcripts.

The Mediator preparation we have used so far was purified from the 0.85 M KCl P11 fraction of the nuclear extract derived from HeLa cells expressing 3xFLAG-tagged MED10 (Nut2) [10]. This form of Mediator is designated simply as Mediator in this study except in Figure 3, where it is designated as Med(0.85) to distinguish it from another form of Mediator, Med(0.5). Med(0.5) was purified from the 0.5 M P11 fraction (Figure 3A), and displayed a subunit composition similar to Med(0.85) with some additional polypeptides. Indeed, Western blot analyses indicated that Med(0.5) contained CDK8, Cyclin C, MED12 and MED13, four subunits that constitute the CDK8 module whereas Med(0.85) lacked any of these subunits (Figure 3B). Both Med(0.85) and Med(0.5) were found to contain Med17, a subunit common to various mediator complexes [33], [34] (Figure 3B). When both forms of Mediator were tested in *in vitro* transcription assays, Med(0.5) was less active than Med(0.85) in enhancing both initiation and reinitiation regardless of the presence of hnRNP R (Figure 3C, left panel, lanes 2 and 3 versus lanes 4 and 5), in agreement with the negative role of the CDK8 module in transcription. When the ratios of reinitiation versus initiation were quantified, Med(0.5) and hnRNP R did not increase the ratio significantly (Figure 3C, bottom panel on the right, lanes 1 and 3). By contrast, Med(0.85) and hnRNP R not only increased the absolute levels of initiation and reinitiation (Figure 3C, top and middle panels on the right, compare lanes 1 and 5), but also increased the ratio of reinitiation versus initiation noticeably (Figure 3C, bottom panel on the right, compare lanes 1 and 5, p = 0.011). These results indicate that Mediator lacking the CDK8 module cooperates efficiently with hnRNP R to facilitate transcription reinitiation *in vitro*; however, the CDK8 module reduced the transcriptional activity of Mediator as well as the cooperativity of Mediator and hnRNP R to facilitate reinitiation.

### Interaction of hnRNP R with Mediator, TFIIB and TBP as well as the 390-nt RNA produced from the G-free cassette

**Figure 4:**
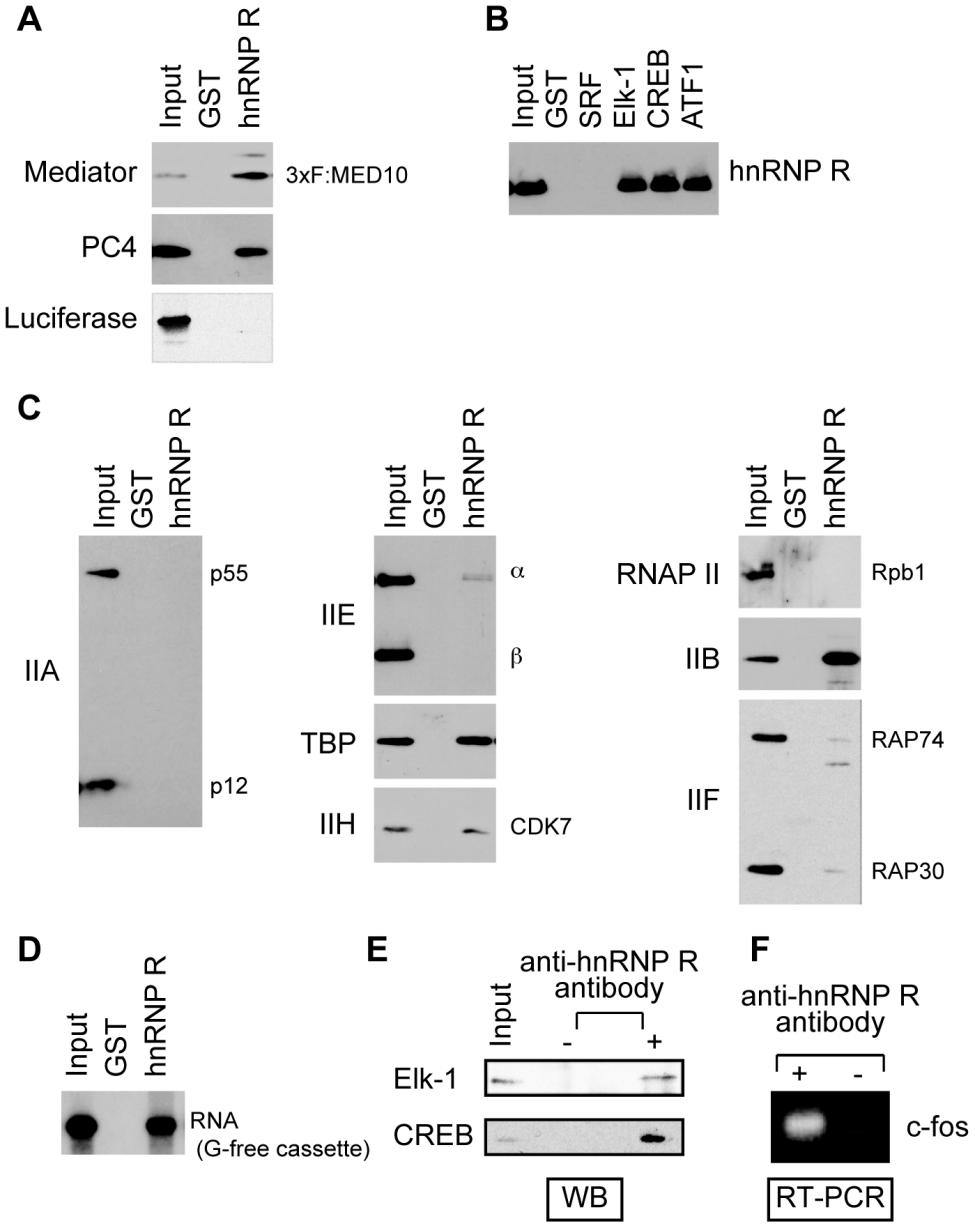
hnRNP R interacts with Mediator and other PIC components, as well as with the transcript RNA. GST pull-down assays were performed to test interaction between hnRNP R and Mediator or PC4 (A), interactions between hnRNP R and the activators (B) and interactions between hnRNP R and GTFs or Pol II (C). (D) The ^32^P-labeled transcript RNA derived from the 390-nt G-free cassette was used to test interaction between hnRNP R and the transcript RNA. GST alone (A–D) and FLAG-tagged luciferase (A) were used as negative controls. (E) Immunoprecipitation was performed using HeLa nuclear extract with the anti-hnRNP R antibody. The proteins precipitated together with hnRNP R were analyzed by western blotting (WB) using anti-Elk-1 and anti-CREB antibodies. (F) RNA was extracted from the immunoprecipitate from HeLa nuclear extract with or without the anti-hnRNP R antibody, and then amplified by RT-PCR and analyzed on a 1% agarose gel.

To explore the underlying mechanisms by which hnRNP R and Mediator enhance c-*fos* transcription, we tested if hnRNP R interacts with activators (SRF, Elk-1, CREB and ATF1), coactivators and the basal transcriptional machinery. As shown in Figure 4A, both Mediator and PC4 interacted with immobilized GST-hnRNP R, but not with GST alone, consistent with the cooperativity of hnRNP R with Mediator and PC4 to enhance c-*fos* transcription [10]. The interactions of hnRNP R with Mediator and PC4 are specific because GST-hnRNP R did not show any interaction with luciferase. In agreement with its role as a transcriptional coactivator, hnRNP R also interacted with immobilized GST-Elk-1, -CREB and -ATF1 but not with GST-SRF or GST (Figure 4B). In transcription reinitiation, Scaffold (TFIIA, TFIID, TFIIE, TFIIH and Mediator) serves as a reinitiation intermediate and recruits Pol II, TFIIF and TFIIB to start the next round of transcription [18]. Consistent with its role in facilitating transcription reinitiation, hnRNP R interacted with some of the factors that constitute Scaffold (TBP, TFIIH and Mediator) as well as with TFIIB, which plays a critical role in recruiting Pol II/TFIIF to the promoter [35]. The results suggest possible protein-protein interactions that may allow hnRNP R to serve as a bridging factor between an incoming Pol II/TFIIF/TFIIB complex and the Scaffold bound on the promoter.

hnRNP R possesses three RRMs and the RGG domain [10], which are found in diverse kinds of RNA-binding proteins [36], [37]. Both domains are generally believed to interact with RNA, but their roles in protein-protein interactions have been also reported [38]. Although hnRNP R can be coprecipitated with the c-*fos* mRNA from the retinal cells [39], it has not been established if hnRNP R binds RNA directly. To formally determine this, we created a plasmid that harbors the T7 promoter immediately upstream of the 390-nt G-free cassette, and used T7 RNA polymerase to produce ^32^P-labeled transcript. The transcript was then incubated with GST-hnRNP R immobilized to Glutathione Sepharose, and after extensive washing as described in the “Materials and Methods”, the bound RNA was isolated and separated on a denaturing polyacrylamide gel. As shown in Figure 4D, the 390-nt transcript bound GST-hnRNP R but not GST alone, demonstrating that hnRNP R directly binds the transcript produced from the G-free cassette under the tested condition. To demonstrate the interactions between the activators and hnRNP R *in vivo*, hnRNP R was immunoprecipitated from HeLa cell extract and the immunoprecipitates were tested for the presence of the activators using antibodies to each activator. As shown in Figure 4E, Elk-1 and CREB were co-immunoprecipitated with hnRNP R, indicating that they interact with hnRNP R in cells. The transcript RNA from the *c-fos* gene is also contained in the immunoprecipitate with the anti-hnRNP R antibody but not that without the antibody, indicating that hnRNP R interacts with the *c-fos* transcript in cells (Figure 4F).

### Reinitiation mediated by hnRNP R is influenced by the length of the G-free cassette

**Figure 5:**
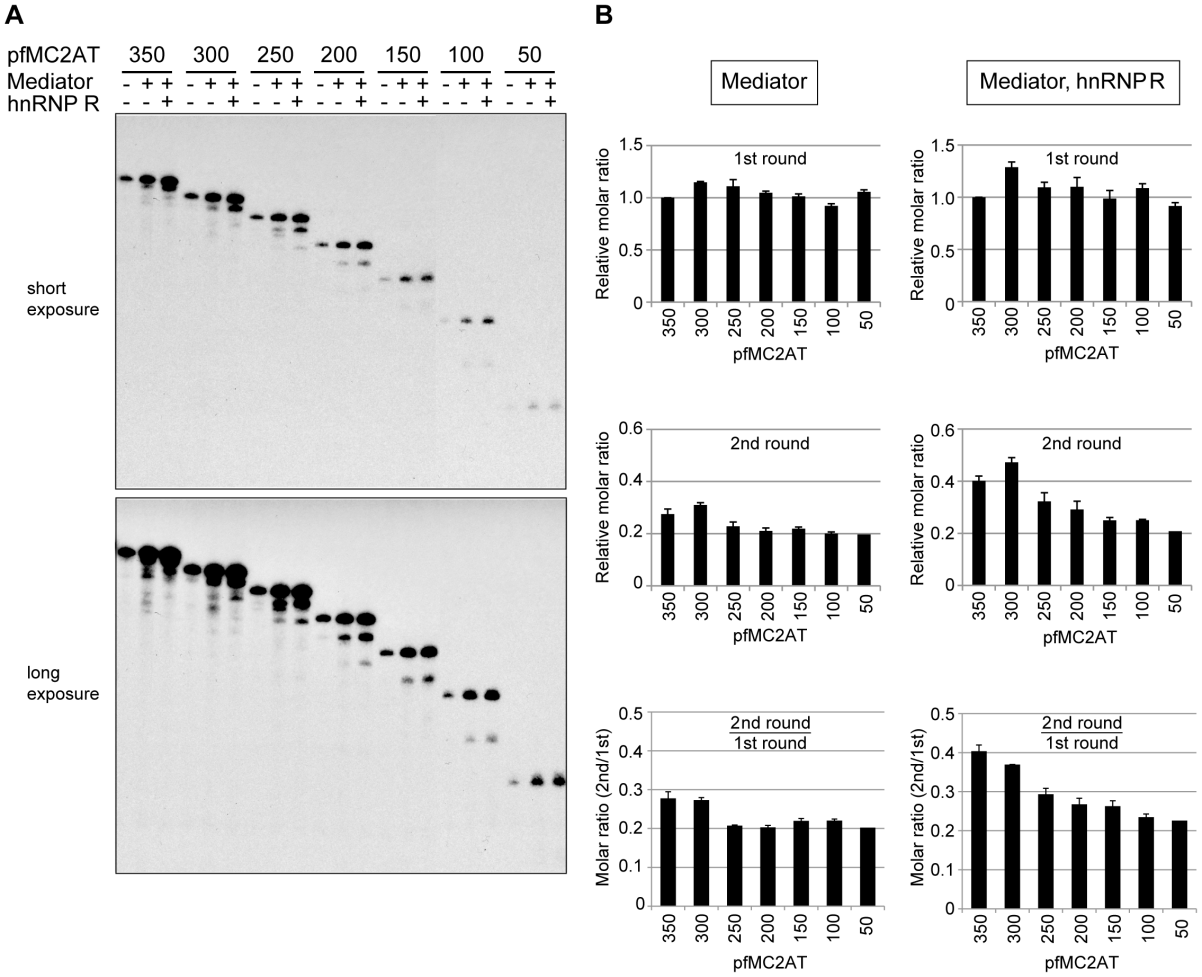
The efficiency of transcription reinitiation depends on the length of the transcripts. (A) *In vitro* transcription was performed using the pfMC2AT templates harboring a G-free cassette that ranges from 350 to 50 nt in length. The numbers above each lane indicate the length of the G-free cassette. Mediator and hnRNP R were added as indicated in the figure. A representative data of three experiments is shown. (B) Quantification of the first-round transcripts and the second-round transcripts in the presence of Mediator alone (“Mediator ONLY”) or both Mediator and hnRNP R (“Mediator(+), hnRNP R(+)”). To obtain the relative molar ratios of the transcripts, the measured transcript levels were normalized by the number of C residues within the transcribed region because [α-^32^P] CTP was used for labeling the transcripts. The molar ratios of the second-round transcripts to the first-round transcripts were calculated using the normalized transcript levels. All the values were averaged from three independent experiments, and error bars represent S.E. The number below each lane indicates the length of the G-free cassette. The p values of the molar ratio (2^nd^/1^st^) on each template versus that on the pfMC2AT(350) are as follows. In the presence of only Mediator: p = 0.83 (pfMC2AT300), p = 0.21 (pfMC2AT250), p = 0.16 (pfMC2AT200), p = 0.13 (pfMC2AT150) and p = 0.13 (pfMC2AT100). In the presence of both Mediator and hnRNP R: p = 0.34 (pfMC2AT300), p = 0.042 (pfMC2AT250), p = 0.018 (pfMC2AT200), p = 0.008 (pfMC2AT150) and p = 0.022 (pfMC2AT100).

To gain further insight into the mechanistic role of hnRNP R in facilitating reinitiation, we used a series of templates that possess the G-free cassette of varying lengths, and analyzed the levels of initiation and reinitiation. All transcription reactions contained Pol II, GTFs, the activators and PC4 whereas Mediator and hnRNP R were added as indicated in Figure 5A. As shown in Figure 5A and quantified in Figure 5B, the levels (in moles) of the initiation were almost invariable regardless of the lengths of the G-free cassette in the presence of Mediator alone (Figure 5B, top panel on the left) or both Mediator and hnRNP R (Figure 5B, top panel on the right), indicating that the length of the G-free cassette does not influence the efficiency of PIC formation, as can be estimated by the levels of initiation. By contrast, the level of reinitiation appeared to correlate with the length of the G-free cassette, especially in the presence of both Mediator and hnRNP R (Figure 5B, middle panel on the right). In fact, when both Mediator and hnRNP R were present in the reactions, there was a clear correlation between the length of the G-free cassette and the ratio of reinitiation to initiation (Figure 5B, bottom panel on the right), indicating that the templates with a longer G-free cassette facilitate reinitiation better than those with a shorter one. Interestingly, this correlation was largely absent in the presence of Mediator alone (Figure 5B, bottom panel on the left). These results indicate that the length of either the G-free cassette or the transcript influences the level of reinitiation when both Mediator and hnRNP R are present.

### Distinct domains of hnRNP R are required for initiation and reinitiation of transcription

**Figure 6:**
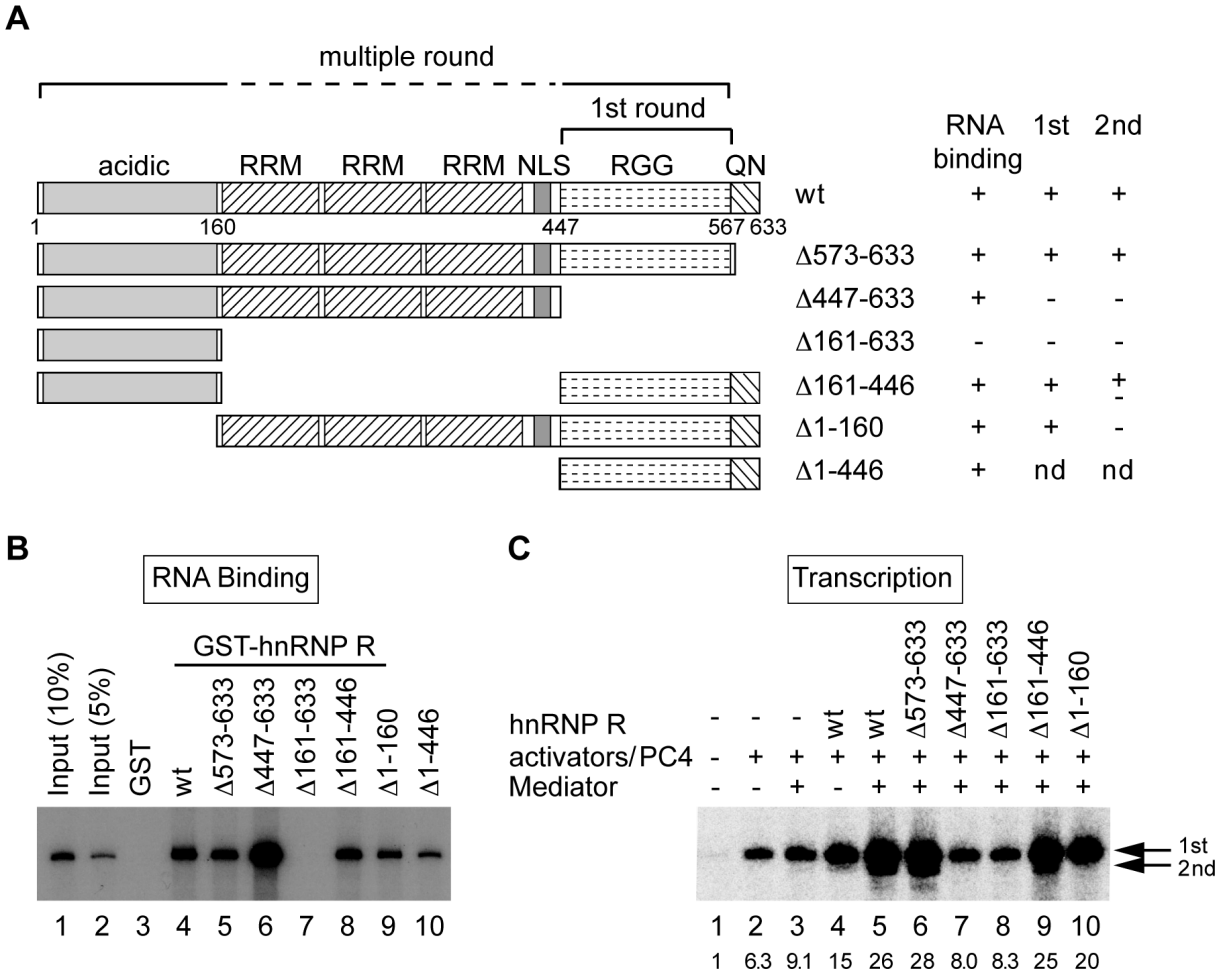
Functional domains of hnRNP R required for facilitating initiation and reinitiation. (A) Schematic diagram of the domain structure of hnRNP R and its deletion mutants are shown. hnRNP R is comprised of the acidic domain (acidic), three RNA recognition motifs (RRMs), a nuclear localization signal (NLS), arginine-glycine-glycine-rich domain (RGG) and the glutamine-asparagine-rich domain (QN). The results of RNA binding assays (shown in B) and *in vitro* transcription (shown in C) for the mutants are summarized on the right. An *in vitro* transcription assay for Δ1-446 was not done (nd) because the mutant was insoluble unless expressed as a GST-fusion protein. The domains required for initiation (1st round) and reinitiation (multiple round) are indicated above the diagram. (B) Binding of the hnRNP R to the RNA derived from the 390-nt G-free cassette was assayed using immobilized GST (lane 3), GST-hnRNP R (lane 4), or GST-hnRNP R mutants (lanes 5–10). The bound RNA was analyzed by electrophoresis and autoradiography, and lanes 1 and 2 contained 10% and 5% of the input RNA. (C) *In vitro* transcription was preformed using the purified FLAG-tagged hnRNP R mutants. pfMC2AT harboring the 390-nt G-free cassette was used as a template. The positions of the first-round and second-round transcripts are indicated by arrows on the right, and the relative levels of the first-round transcript are indicated below each lane.

To determine the functional domains of hnRNP R required for RNA binding and reinitiation, we created deletion mutants of hnRNP R. First we produced hnRNP R mutants (Figure 6A) as GST-fusion proteins using the baculovirus expression system and tested the binding of the ^32^P-labeled 390-nt transcript to the immobilized GST-hnRNP R mutants. As shown in Figure 6B and summarized in Figure 6A, deletion of QN (Δ573-633) or the acidic domain (Δ1-160) did not affect the ability of hnRNP R to bind the transcript (lane 5 and 9). The mutants lacking either RRMs (Δ161-446) or the RGG domain (Δ447-633) could bind the transcript (lanes 6 and 8); however, the mutant lacking both RRMs and the RGG domain (Δ161-633) could not bind the RNA at all (lane 7). These results indicate that RRMs and the RGG domain can bind RNA by themselves and at least one of the two RNA-binding domains is required for hnRNP R to bind RNA.

Next, to define the regions of hnRNP R required for facilitating initiation and reinitiation, FLAG-tagged hnRNP R mutants were expressed by the baculovirus system and purified from the infected insect cells (supplemental Figure S1). The mutants were then tested in *in vitro* transcription assays using the template containing the 390-nt G-free cassette, which is most efficient in reinitiation. As shown in Figure 6C, deletion of QN (Δ573-633) had no effect on the transcriptional activity of hnRNP R (lane 6), but further deletion of the RGG domain (Δ447-633) or both RRMs and the RGG domain (Δ161-633) completely eliminated the activity of hnRNP R to facilitate both initiation and reinitiation (lanes 7 and 8). Internal deletion of RRMs (Δ161-446) did not affect initiation but reduced reinitiation moderately (lane 9). Interestingly, despite its ability to facilitate initiation as efficiently as wild-type hnRNP R, the deletion mutant of the acidic domain (Δ1-160) failed to facilitate reinitiation at all (lane 10). Thus, the RGG domain is required, and may be sufficient, for facilitating initiation; however, the acidic domain and RRMs are additionally required for facilitating reinitiation efficiently.

### hnRNP R is required for rapid induction of the *c-fos* gene *in vivo*

**Figure 7:**
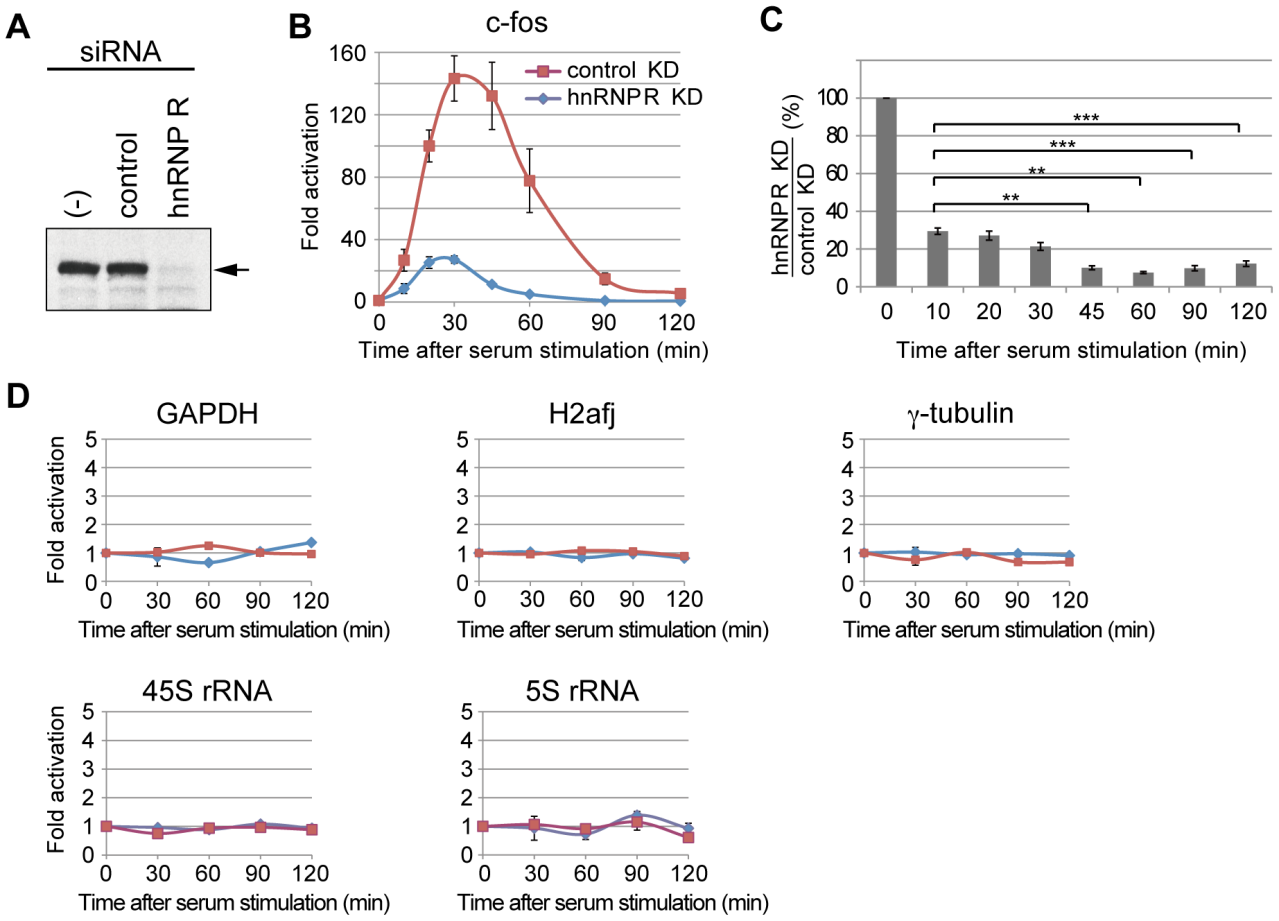
hnRNP R is required for transcriptional induction of the c-fos gene. (A) Mouse C3H10T1/2 cells were treated by siRNA for hnRNP R (hnRNP R) or control siRNA (control), and the levels of hnRNP R were analyzed by Western blotting using an anti-hnRNP R antibody. The cells that were not treated with siRNA are indicated by (-). (B) Total cellular RNAs were prepared from the siRNA-treated cells, and RT-qPCR assays were used to determine the effect of hnRNP R knockdown on the induction of the *c-fos* gene after serum stimulation. The expression levels of the α-tubulin gene were used to normalize those of the *c-fos* gene at each time point, and the expression levels before serum stimulation were used to calculate fold activation at each time point. The values were averaged from five independent experiments, and the error bars represent S.E. The followings are the p values when comparing the *c-fos* expression level after hnRNP R knockdown versus that after control knockdown at each time point: p = 0.028 (10 min), p = 0.006 (20 min), p = 0.007 (30 min), p = 0.012 (45 min), p = 0.049 (60 min), p = 0.05 (90 min) and p = 0.01 (12 0min). (C) The ratios of the c-fos expression level after hnRNP R knockdown against that after control knockdown were calculated. The followings are the p values when comparing the ratio at each time point versus that at 10min: p = 0.404 (20 min), p = 0.107 (30 min), p = 0.001 (45 min), p = 0.002 (60 min), p = 0.0006 (90 min) and p = 0.0002 (120 min). Two asterisks (**) indicate p<0.01 and three asterisks (***) indicate p<0.001. (D) Effects of hnRNP R knockdown on expression of constitutive genes were analyzed. The p values at each time point are shown in the supplemental table S2.

So far, the effect of hnRNP R on transcription has been analyzed solely in biochemical assays. To find if hnRNP R is involved in transcription of the endogenous c-*fos* gene, we knocked down hnRNP R in mouse cells and tested its effects on c-*fos* induction. After C3H 10T1/2 cells were transfected with siRNA or control siRNA, the cells were starved in 0.5% FBS for 24 hrs, and stimulated by 20% FBS. After RNA extraction, the c-*fos* transcript was analyzed by quantitative RT-PCR. Figure 7A shows that the amount of hnRNP R in the cells was reduced to less than 20% of its original level by the specific siRNA but remained unchanged by the control siRNA. In hnRNP R-knockdown cells, the maximum induction of the c-*fos* gene by serum was reduced to ∼30-fold as compared to ∼150-fold in the control cells (Figure 7B), indicating the important role of hnRNP R in c-*fos* induction upon serum stimulation. Moreover, the reduction of the *c-fos* transcript level upon hnRNP R knockdown was more pronounced at later time points (e.g. 45, 60, 90 and 120 min as compared to 10 min after serum induction), where the ratio of the reinitiated transcripts to the first transcript is presumably greater (Figure 7C).

Unlike the *c-fos* gene, constitutive genes such as *GAPDH*, *H2afj* and γ-tubulin were transcribed at essentially the same levels in both hnRNP R-knockdown and control cells (Figure 7D), indicating that hnRNP R is not required for these genes. We also tested the effect of hnRNP R knockdown on transcription of the 45S rRNA and 5S rRNA genes, which are transcribed at high rates by Pol I and Pol III, respectively. Figure 7D shows that transcription levels of both genes, which remained almost invariable upon serum induction, were not affected by hnRNP R knockdown, indicating that hnRNP R is not involved in transcription by Pol I and Pol III.

## Discussion

In this study, we have found that hnRNP R cooperates with Mediator to facilitate transcription reinitiation *in vitro*. A previous study using a yeast transcription system showed that Mediator promotes reinitiation of Pol II transcription [18]. After initiation of transcription, a structure termed Scaffold, which consists of TFIIA, TFIID, TFIIE, TFIIH and Mediator, remains at the promoter; subsequent reentry of Pol II together with TFIIB and TFIIF onto the Scaffold reinitiates the next round of transcription [18]. In rapidly inducible genes, Pol II appears to have initiated transcription and then paused at +25∼+45 before induction [40], indicating that Scaffold rather than the complete PIC is likely to occupy their promoters before induction. Because rates of transcription reinitiation are higher than those of initiation [41], rapidly inducible genes such as *c-fos* may respond to induction mostly by prompting Scaffold to produce the transcripts via transcription reinitiation without undergoing *de novo* PIC formation. In this regard, the significant effect of hnRNP R knockdown on induction of the *c-fos* gene *in vivo* (Figure 7) can be explained, at least in part, by the critical role of hnRNP R in facilitating transcription reinitiation *in vitro* (Figures 1–3). Indeed, hnRNP R knockdown has more pronounced effects at later time points, where a larger fraction of the transcripts is presumed to derive from reinitiation. These effects are also consistent with the role for hnRNP R in enhancing reinitiation.

The potent cooperativity between hnRNP R and Mediator with regards to transcription reinitiation may be accounted for by two possible mechanisms. First, hnRNP R may provide an additional interface that augments interactions between an incoming Pol II/TFIIF/TFIIB complex and Scaffold via interactions of hnRNP R with TFIIB and Scaffold (via TBP, TFIIH and Mediator). Indeed, direct protein-protein interactions of Mediator with TFIIB and Pol II have been shown to be essential both for the formation of the PIC [42] and for reinitiation by Scaffold [18]. Second, given the role of activators in stabilizing the promoter-bound Scaffold during reinitiation [18], the interactions of hnRNP R with activators and Scaffold (Figure 4B) may allow hnRNP R to cooperate with DNA-bound activators to stabilize Scaffold.

Because of its importance as an integrative hub for transcriptional control [7], Mediator would likely to possess multiple regulatory functions. Indeed, one such function resides in a Mediator subcomplex, termed the CDK8 module, which consists of CDK8, Cyclin C, MED12 and MED13 [8]. Knuesel et al. reported that this module represses transcription reinitiation, by using Sarkosyl to indirectly estimate the reinitiation levels [28]. Our results, which directly measured the transcript from reinitiation, not only agree well with their findings but also reveal additional function of the CDK8 module in attenuating the effect on transcription reinitiation by Mediator and hnRNP R. However, substantial evidence also indicates that CDK8, presumably as the CDK8 module, enhances rather than inhibit transcription [29], [30], [31]. It is likely that the CDK8 module is dynamically released from [43] and re-incorporated into the promoter-bound Mediator [28], as exemplified by poly(ADP-ribose) polymerase 1, which displaces the CDK8 module from an inactive PIC that contains Mediator [44]. Such dynamic association could provide a rheostat mechanism [28] by which Mediator and hnRNP R regulate reinitiation in rapidly induced genes.

Additional, but intriguing, insight into how hnRNP R facilitates reinitiation is provided by our observation that the reinitiation level correlates with the length of the transcribed region (Figure 5), an effect that could be attributed to the length of either the template DNA or transcript RNA. In the former possibility, hnRNP R would act as an elongation factor, whose effect is usually more prominent on the longer templates. Indeed, an elongation factor, SII, was reported previously to facilitate transcription reinitiation, which was subsequently shown as a secondary effect from the stimulation of elongation by SII [22], [45]. Two lines of evidence, however, argue against the possibility that hnRNP R functions in a manner similar to SII. First, hnRNP R interacts with activators, TBP and Mediator that remain at the promoter but displays no interaction whatsoever with Pol II (Figure 4). Second, the requirement of distinct domains of hnRNP R for facilitating initiation and reinitiation would be difficult to reconcile with the fact that hnRNP R acts at the elongation step (Figure 6). Thus, we favor the latter possibility that the transcript RNA is somehow involved in enhancing reinitiation. For instance, the transcript RNA from the preceding Pol II could contact hnRNP R via its RNA-binding domains and enhance the functional cooperation of hnRNP R with Mediator for recruiting the next Pol II.

The latter possibility, however, requires that those genes that utilize hnRNP R for facilitating reinitiaion should be loaded with multiple Pol IIs. Most active Class II genes, however, appear to contain only one Pol II within their transcribed regions *in vivo* [16], [46], [47]. Under these circumstances, the transcript from an elongating Pol II is probably released from the gene before the next round of transcription initiates. Some studies, however, showed that certain inducible Class II genes are transcribed simultaneously by multiple Pol IIs when they are maximally induced [48], [49], [50]. In these genes, it would be possible that the transcript from an elongating Pol II can influence the reinitiation of the next Pol II. Because the c-fos gene is but one of the immediate-early genes that are induced rapidly by serum stimulation, a genome-wide analysis is in progress to see if hnRNP R is more generally required for rapid induction of gene transcription.

Finally, *in vitro* transcription reactions using nuclear extract or crude fractions produce several reinitiations [22], [24], [45], as opposed to a few reinitiations observed in our purified transcription system. This suggests the existence of additional factors that facilitate transcription reinitiation. Identification of such factors in the future studies will help us understand how genes respond to activating signals by regulating transcription reinitiation.

## Supporting Information

Figure S1 **Purified deletion mutants of hnRNP R.** The FLAG-tagged mutants of hnRNP R were expressed in High Five insect cells using the baculovirus system. The mutants were purified first by HiTrap SP HP and HiTrap Q HP, and then by affinity purification using anti-FLAG® M2-Agarose (Sigma-Ardrich). 300 ng each of the purified mutants were separated on a 12% SDS-polyacrylamide gel and stained with Coomassie Brilliant Blue R-250.
(TIF)

Table S1 **Primers used for quantitative RT-qPCR to analyze the expression levels of the c-fos, GAPDH, H2afj, gamma-tubulin, 45S rRNA, 5S rRNA and alpha-tubulin genes.**
(DOC)

Table S2 **The p values for the RT-qPCR analyses shown in** **Figure 7D** **.** The table shows the p value when comparing the expression levels of the constitutive genes before and after hnRNP R knockdown at each time point.
(DOCX)
