# Supplementary figures and images for: Heterogeneous Nuclear Ribonucleoprotein R Cooperates with Mediator to Facilitate Transcription Reinitiation on the c-Fos Gene
Source: PLoS One. 2013 Aug 13;8(8):e72496. doi: 10.1371/journal.pone.0072496 (PMC3742609; doi:10.1371/journal.pone.0072496)

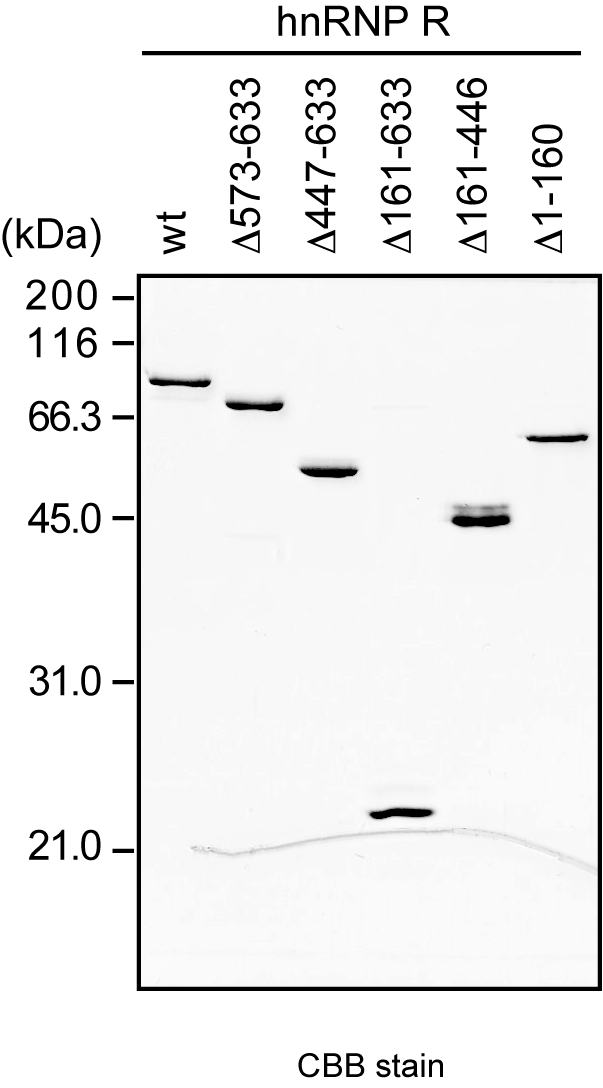

Supplement: Figure S1 — Purified deletion mutants of hnRNP R. The FLAG-tagged mutants of hnRNP R were expressed in High Five insect cells using the baculovirus system. The mutants were purified first by HiTrap SP HP and HiTrap Q HP, and then by affinity purification using anti-FLAG® M2-Agarose (Sigma-Ardrich). 300 ng each of the purified mutants were separated on a 12% SDS-polyacrylamide gel and stained with Coomassie Brilliant Blue R-250. (TIF) [file pone.0072496.s001.tif]
