# Supplementary material for: Heterogeneous Nuclear Ribonucleoprotein R Cooperates with Mediator to Facilitate Transcription Reinitiation on the c-Fos Gene
Source: PLoS One. 2013 Aug 13;8(8):e72496. doi: 10.1371/journal.pone.0072496 (PMC3742609; doi:10.1371/journal.pone.0072496)
Supplement: Table S1 — Primers used for quantitative RT-qPCR to analyze the expression levels of the c-fos, GAPDH, H2afj, gamma-tubulin, 45S rRNA, 5S rRNA and alpha-tubulin genes. (DOC) [file pone.0072496.s002.doc]

**Table S1.** Primer sets used for RT-qPCR.

| *c-fos* | 5’ primer | GAGGACCTTACCTGTTCGTGAAA |
| --- | --- | --- |
|  | 3’ primer | CCAGATGTGGATGCTTGCAA |
| *GAPDH* | 5’ primer | CCTGCTCCCCCTACACACA |
|  | 3’ primer | CCTGTTCTTCTCGGGCAAAA |
| *H2afj* | 5’ primer | CGTCCTGCCCAATATCCAG |
|  | 3’ primer | TCTGCACCCGTCTGTCG |
| *gamma- tubulin* | 5’ primer | CGGACCTGTCGCCAGTTT |
|  | 3’ primer | TGCGGAACTGCTCCATGA |
| *45S rRNA* | 5’ primer | CCTTTAACGAGGATCCATTGGA |
|  | 3’ primer | CGCTATTGGAGCTGGAATTACC |
| *5S rRNA* | 5’ primer | CTCGTCTGATCTCGGAAGCTAAG |
|  | 3’ primer | GGCGGTCTCCCATCCAAGTA |
| *alpha- tubulin* | 5’ primer | GGTTCCCAAAGATGTCAATGCT |
|  | 3’ primer | CAAACTGGATGGTACGCTTGGT |
