# Supplementary material for: Heterogeneous Nuclear Ribonucleoprotein R Cooperates with Mediator to Facilitate Transcription Reinitiation on the c-Fos Gene
Source: PLoS One. 2013 Aug 13;8(8):e72496. doi: 10.1371/journal.pone.0072496 (PMC3742609; doi:10.1371/journal.pone.0072496)
Supplement: Table S2 — The p values for the RT-qPCR analyses shown in Figure 7D . The table shows the p value when comparing the expression levels of the constitutive genes before and after hnRNP R knockdown at each time point. (DOCX) [file pone.0072496.s003.docx]

**Table S2.** P values for the RT-qPCR data in Figure 7D.

|  | 30min | 60min | 90min | 120min |
| --- | --- | --- | --- | --- |
| GAPDH | 0.201 | 0.320 | 0.859 | 0.509 |
| H2afj | 0.661 | 0.297 | 0.493 | 0.760 |
| γ-tubulin | 0.161 | 0.721 | 0.142 | 0.363 |
| 45S rRNA | 0.462 | 0.822 | 0.770 | 0.827 |
| 5S rRNA | 0.871 | 0.601 | 0.292 | 0.539 |
